# Loss of work-related income impoverishes people with SCI and their families in Bangladesh

**DOI:** 10.1038/s41393-019-0382-1

**Published:** 2019-11-26

**Authors:** Mohammad Sohrab Hossain, Lisa A. Harvey, Md. Shofiqul Islam, Md. Akhlasur Rahman, Hueiming Liu, Robert D. Herbert, on behalf of the CIVIC Trial Collaboration

**Affiliations:** 1 Centre for the Rehabilitation of the Paralysed, Savar, Dhaka Bangladesh; 2 John Walsh Centre for Rehabilitation Research, Kolling Institute, Sydney Medical School/Northern, University of Sydney, St Leonards, NSW Australia; 3 George Institute for Global Health, Sydney, NSW Australia; 4 Neuroscience Research Australia, Barker Street, Randwick, NSW 2031 Australia

**Keywords:** Neurology, Risk factors

## Abstract

**Study design:**

Cross-sectional study.

**Objectives:**

To determine the degree of impoverishment of people with spinal cord injury (SCI) and their families in Bangladesh caused by loss of work-related income following injury.

**Setting:**

Spinal cord injury centre, Bangladesh.

**Methods:**

A total of 410 wheelchair-dependent people with recent SCI about to be discharged from a hospital in Bangladesh were interviewed to determine the size of their families, their incomes from paid work prior to injury and the incomes of their family members. These data were used to calculate income per family unit and per family member prior to and immediately after injury.

**Results:**

Ninety percent of the participants were men, 98% were from rural areas of Bangladesh and 58% were manual labours prior to injury. Median (interquartile range, IQR) family size was 5 (4–6) people. Prior to injury, 74% of participants were the main income earners for their families and 50% provided the only source of income for their families. Participants’ median (IQR) monthly income prior to injury was US$106 (US$60–US$180) per person and family members’ income was US$30 (US$19–US$48) per person. After injury, the median income (IQR) of each family member dropped to US$0 (US$0–US$18) placing 91% of families below the extreme poverty line of US$37.50 per person per month (equivalent to US$1.25 per day).

**Conclusion:**

In Bangladesh, SCI have profound financial implications for individuals and their families and causes extreme poverty. This is because those most often injured are young and the main income earners for their families.

## Introduction

In Bangladesh, spinal cord injuries (SCI) are commonly due to work-related accidents in young manual labourers [1–5], often with low socioeconomic backgrounds [6–8]. A similar pattern is seen across other low- and middle-income countries (LMIC) [9–12]. SCI causes hardship for those who are injured and often also for the families who are financially dependent on them [3, 13–15]. The loss of income and additional medical and ongoing costs associated with the injury place a large financial strain on families [16, 17]. Not surprisingly, therefore, SCI in Bangladesh and other LMIC often throws families into extreme poverty [18–20].

We previously quantified the financial implications of SCI on individuals and their families in Bangladesh [3]. For that purpose, we interviewed 283 of the 350 people with an SCI who had been discharged from a large hospital in Bangladesh in 2011, 2 years following discharge (55 of the 350 had died and 11 were lost to follow-up). Interviewees were asked about family income and employment status prior to injury and at the time of interview. We found that the median (interquartile range, IQR) income of each family member living with a person with SCI was US$20 (US$13–US$39) per person per month. This is less than the extreme poverty line in Bangladesh (US$37.50 per person per month or US$1.25 per person per day) [21]. These data probably underestimate the financial distress experienced by many people with SCI and their families because the cohort included 115 people who were walking and hence less disabled than their wheelchair-dependent counterparts.

In a 5-year follow-up of the same cohort we collected and analysed additional data according to walking status at discharge. The median (IQR) income of those who were wheelchair dependent at discharge and still alive at 5 years (*n* = 141) was US$0 (US$0–US$65); much less than pre-injury incomes of US$65 (US$39–US$104). Seventy-five percent were living below the poverty line (US$57 per month or US$1.90 per day) and only 35% were in full-time employment 5 years after discharge. The limitation of these data is that we did not calculate income per family member, and the pre-injury data may not have been accurate because we asked participants to remember their incomes many years earlier. In addition, we did not have data about participants’ and their families’ financial situation during the period after injury when the person was hospitalised. The current study was designed to overcome these limitations and provide more accurate data on the financial implications of SCI on those injured and their families in Bangladesh. Specifically, the aim was to determine the degree of impoverishment of people with SCI and their families caused by the loss of work-related income following SCI in Bangladesh.

The data from this study reflects those with recent SCI admitted to the Centre for the Rehabilitation of the Paralysed (CRP) who were wheelchair dependent on discharge. The CRP is a large rehabilitation centre in Bangladesh that admits more than 400 people with recent SCI from anywhere in Bangladesh each year (see [3] for the details about the types of people typically admitted each year to CRP). People with SCI are referred to CRP by government and non-government hospitals, although some patients are also self-referred. Those with recent SCI are given priority admission over people seeking readmission for the management of pressure ulcers or other problems that develop after discharge from CRP. The CRP provides treatment, and multi-disciplinary comprehensive rehabilitation free of charge unless the patient has some capacity to contribute to the cost. It is the only rehabilitation facility specifically for people with SCI in Bangladesh and is recognised as one of the biggest centres of its kind in Asia.

## Method

This study is part of an ongoing randomised clinical trial. The trial, known as the CIVIC trial, is due for completion in February 2020. It will determine the effect of community-based care compared with usual care on 2-year survival [22]. In the current paper we present an analysis of some descriptive data collected prior to randomisation. The trial was registered with Australian New Zealand Clinical Trials Registry (ACTRN12615000630516) and Universal Trial Number (U1111‐1171‐1876). The institutional and governmental regulations concerning the ethical use of human volunteers were followed.

### Participants

Five hundred and nine people who were about to be discharged from CRP in Dhaka, Bangladesh, were screened between July 2015 and March 2018 for inclusion. Those who met the inclusion criteria and provided consent were recruited to the CIVIC trial. Participants were eligible if they were more than 16 years of age, had sustained a traumatic or nontraumatic SCI within the last 2 years and required a wheelchair daily for mobility. Potential participants were excluded if they were planning to move to another country or were being transferred to another hospital for medical care. Sixty-six people did not meet the inclusion criteria, and 33 were not randomised because either they declined to participate in the trial (*n* = 24) or were discharged unexpectedly (*n* = 9). Ultimately, 410 people were randomised to groups and therefore participated in the study.

### Data collection

Data were collected in face-to-face interviews conducted prior to randomisation and discharge using standardised forms. Participants were asked in Bangla about the number of family members (adults and children) living with them as a family unit. We did not define the age of a child but most in Bangladesh assume that a child is a person aged less than 14 years. Participants were asked to identify the main income earner for the family. They were also asked about the employment status and income of all family members. Financial data were recorded in local currency (BDT) and subsequently converted to United States Dollar (US$) using the conversion rate at www.xe.com (accessed on 15^th^ April 2019). Data were also collected on participants’ places of residence (rural versus urban), type of work prior to injury and literacy levels to gauge socioeconomic backgrounds.

### Data analysis

The total income for each family was divided by the number of adults and children in the family to derive income per family member. Families were then defined as living below the poverty line or extreme poverty line on the basis of the average income per family member. The poverty line was defined as US$57 per person per month (~US$1.90 per person per day) and the extreme poverty line was defined as US$37.50 per person per month (~US$1.25 per person per day) as per the definitions of the United Nations and World Bank [21, 23–25].

The same calculations were repeated but with the income of the person with SCI removed to determine the average income for each adult and child within a family after loss of income. These later calculations assumed that the person with SCI had no income once injured and while in hospital, and that there was no change in the employment status of other family members since injury.

## Results

### Participants’ characteristics

The characteristics of participants and their families as well as their work status are shown in Tables 1 and 2. The median (IQR) age of participants was 33 years (25–43), 90% were male, 57% had paraplegia, 43% had tetraplegia, 69% were married and 71% had American Spinal Injuries Association (ASIA) Impairment Scale A lesions. In addition, 98% were from rural areas of Bangladesh and 65% had no or limited ability to read. The median (IQR) size of each family (including the person with SCI) was 5 (4–6) individuals with a median (IQR) 2 (1–3) adults and 2 (1–2) children per family. We did not ask participants to specify their relationship with other adults in the household but they could have been spouses, parents, siblings or children.

**Table 1:** Characteristics of participants

Characteristics	Total
Participants, (*n*)	410
Gender, *n* (%)	
Male	369 (90%)
Female	41 (10%)
Age (years), median (IQR)	33 (25–43)
Time since injury (months), median (IQR)	6 (5–8)
Time in CRP (months), median (IQR)	4 (4–6)
Marital Status, *n* (%)	
Married	284 (69%)
Not married	107 (26%)
Separated/divorced	15 (4%)
Widowed	4 (1%)
Geographic location*, *n* (%)	
Dhaka	126 (30%)
Rajshahi	43 (11%)
Chittagong	74 (18%)
Sylhet	19 (5%)
Khulna	62 (15%)
Barisal	36 (9%)
Rangpur	17 (4%)
Mymensingh	33 (8%)
Residency before injury, *n* (%)	
Rural	400 (98%)
Urban	10 (2%)
ASIA impairment scale (AIS), *n* (%)	
A	292 (71%)
B	57 (14%)
C	55 (13%)
D	6 (2%)
Type of injury, *n* (%)	
Paraplegia	235 (57%)
Tetraplegia	175 (43%)
Cause of injury, *n* (%)	
Traumatic	390 (95%)
Nontraumatic	20 (5%)
Ability to read and write Bangla, *n* (%)	
Good ability	146 (35%)
Limited ability	143 (35%)
No ability	121 (30%)

^*^These are Bangladeshi divisions that include cities and large surrounding rural areas

**Table 2:** The work status and size of participants’ (*n* = 410) families prior to injury

**Family details**	
Size of families, median (IQR)	5 (4–6)
Number of children in each family, median (IQR))	2 (1–2)
Number of adults in each family, median (IQR)	2 (1–3)
Total number of family members including participants, *n*	2212
**Paid work status of participants at time of injury**	
Full-time employment (>30 h per week), *n* (%)	335 (82%)
Part-time employment (<30 h per week), *n* (%)	10 (2%)
**Unpaid work status of participants at time of injury**	
Home duties, *n* (%)	20 (5%)
Student, *n* (%)	38 (9%)
Other, *n* (%)	7 (2%)
**Types of work**	
Manual labourers (light and heavy)	200 (58%)
Small business	43 (12%)
Tradesperson	60 (17%)
Office worker	23 (7%)
Shopkeeper	10 (3%)
Professional	9 (3%)
Number of families in which participant was the only income earner, *n* (%)	206 (50%)
Number of families in which participant was the main income earner, *n* (%)	302 (74%)

### Income

Prior to injury, 82% of participants were in full-time employment. Most participants (58%) were manual labourers or tradespersons (17%) prior to injury, and only nine participants (3%) had professional jobs. The others were either office workers, business workers or shopkeepers. Seventy-four percent were the main income earners for their families. In 50% of families, no other person worked. The incomes of participants and their family members before and after SCI are shown in Table 3. The monthly median (IQR) income of each family member prior to injury was US$30 (US$19–US$48) per person. This was sufficient to keep 33% of family members above the extreme poverty line. After injury, the monthly median (IQR) income of each family member was US$0 (US$0–US$18) per family member. This was sufficient to keep 9% of family member above the extreme poverty line. That is, after injury, 91% of family members were living below the extreme poverty line of US$37.50 per person per month.

**Table 3:** The financial situation of participants (*n* = 410), households and family members* before and after injury

	Before injury	After injury
**Income (US$ per month; median and IQR)**		
Participants	106 (60–180)	–
Households	156 (96–240)	0 (0–102)
Family members	30 (19–48)	0 (0–18)
**Living below poverty line (*n*, %)** (less than US$57 per month or US$1.90 per day)		
Participants	86 (21%)	410 (100%)
Households	329 (80%)	397 (97%)
Family members	1833 (83%)	2160 (98%)
**Living below extreme poverty line (*n*, %)** (less than US$37.50 per month or US$1.25 per day)		
Participants	76 (19%)	410 (100%)
Households	262 (64%)	372 (91%)
Family members	1473 (67%)	2018 (91%)

^*^Including participants (*n* = 2212)

## Discussion

This study investigated work-related family incomes before and immediately after SCI in Bangladesh with the aim of determining the degree of impoverishment of people with SCI and their families. A notable finding was just how impoverished families were prior to injury: families had a median (IQR) income of US$30 (US$19–US$48) per family member per month. Thus 67% of family members were living below the extreme poverty line prior to injury. It was not surprising to see the median (IQR) income drop to US$0 (US$0–US$18) per month per family member following injury, because those injured where typically young males and 74% were the main income earners for their families. This drop of income placed 91% of families below the extreme poverty line. There are various ways of defining poverty with poverty lines often adjusted for the number of children under the age of 12 years living in a family unit. However, regardless of how poverty is defined, there can be little doubt that our findings highlight the dire financial implications that SCI can have on families in Bangladesh.

Our data on participants’ incomes prior to injury are broadly consistent with data from our previous study of a similar cohort discharged from CRP in 2011 [26]. We found in our previous study that participants who were wheelchair dependent at discharge earnt a median (IQR) of US$65 (US$39–US$104) per month prior to injury. In the current cohort, participants earnt a median (IQR) of US$106 (US$60–US$180) prior to injury. The higher income observed in the current study may reflect wage inflation over the intervening 4 years (estimated to be 5.75% per year) [27]. Nonetheless, the incomes of participants of both cohorts were lower than in the general population of Bangladesh. For example, while 67% and 83% of family members in the current cohort lived below the extreme poverty and poverty lines prior to injury, respectively, only 13% and 23% of the general population of Bangladesh are in the same financial situations [13, 28–30]. Similarly, the literacy levels of our cohort (only 35% had a good ability to read and write) were lower than the national literacy rate of 74% [31]. These factors together point to the social disadvantage of our cohort, and probably reflect that those most likely to sustain an SCI in Bangladesh are unskilled labourers from poor rural backgrounds [6, 32].

Our data are not necessarily reflective of all people who sustain an SCI in Bangladesh. Bangladesh has a population of 164 million. While there are no accurate data on the incidence of SCI in Bangladesh, it is likely that the incidence is between 20 and 40 per million [8, 33]. This equates to between 3280 and 6560 people with a new SCI each year. The CRP only admits 400 people with recent SCI per year. So, clearly, many people who sustain an SCI are not admitted to CRP and these people may be different to those that are. For example, those from higher socioeconomic backgrounds may be admitted to private hospitals or hospitals in other countries. In contrast, those from even poorer backgrounds than our cohort may never get to CRP or never get to any hospital because of economic and social disadvantage.

Our calculations may underestimate the extent of the financial strain placed on families because we did not consider additional costs incurred by families for healthcare services. The medical costs for our participants were largely covered by CRP but nonetheless some participants may have incurred health-related costs before they were admitted to CRP. The situation may be quite different in other LMICs or other parts of Bangladesh. For example, one study from Nigeria found that 41% of participants used 50% of their annual income to meet the acute medical costs of an injured person [17]. This does not include the ongoing costs incurred after discharge from hospital.

Financial hardship following SCI affects all aspects of life and contributes to premature death in both high-income countries and LMIC [34, 35]. Our studies in Bangladesh indicate that 32% of participants who are wheelchair dependent at discharge die within 5 years, primarily from pressure ulcers [36]. The causal links between financial hardship, pressure ulcers and premature death is probably complex but it is plausible that poverty plays a major role.

A reduction in poverty needs to be a major focus of future initiatives to improve the lives of people with SCI and their families in LMIC [16, 37]. The data provided in this study could be used to highlight the financial hardship of people with SCI and their families, and to encourage governments to provide some financial support. In addition, attention needs to continue to be directed at getting those injured back to work. For this purpose, vocational training needs to be at the centre of any rehabilitation program [13, 17]. A study from Nepal found that less than half of those with an SCI had any income many years after discharge from hospital [38]. Our previous study  from Bangladesh showed that only 37% of people who were wheelchair dependent on discharge from hospital were employed 2 years after discharge [3] and only 42% were employed 6 years after discharge [26]. Some evidence suggests that those who do gain employment have much lower salaries than they had prior to injury [38].

Vocational training is provided at CRP but unfortunately many barriers to employment need to be overcome. These include wheelchair-inaccessible environments, and the low literacy rates and skill levels of people who are typically injured (see Tables 1 and 2). In addition, discrimination and poor societal attitudes to those with disabilities are still widespread. Often, the best employment option for people with SCI and limited skills is to set up small businesses or shops [16]. This may require a small amount of initial financial assistance. It is also important that young students who are injured are supported to return to their education. In addition, attention could be directed at vocational training for the wives of injured men. The women may require more support than in most countries because in Bangladesh women do not typically work outside the home [6]. However, the employment of wives could help relieve the financial pressures on families.

There are several limitations to this study. The main limitation, as discussed above, is that we cannot generalise our results to all people who sustain SCI in Bangladesh or other LMIC. The second limitation is that while our cohort is largely reflective of those admitted to CRP who survive to discharge and are wheelchair dependent, we were unable to recruit 33 potentially eligible participants (9% of the potentially eligible cohort). This may have introduced a small selection bias. Thirdly, we did not verify what participants told us at the time of discharge about their and their families’ income and employment status prior to injury. Participants may not have accurately remembered, or may not have known, or may have perceived that it was within their interests to underestimate their family’s incomes. In addition, we did not capture people’s assets or money given to people by extended family members or friends. We did not explore families’ capacities to be self-sufficient through their own crops and animals. Nor did we ask participants about other potential sources of income from insurance, government, prior savings or other sources, although these sources of income are uncommon in Bangladesh particularly for those working in unskilled jobs. We know from anecdotal evidence that many people sell property and animals or take loans to support their families. The results of this study should therefore be seen within these limitations. Future studies could better explore how families survive on such little income.

In summary, SCI have profound financial implications for individuals and their families, and cause extreme poverty in Bangladesh. This is because those most often injured are young people from low socioeconomic backgrounds who are the main income earners for their families.

## Data Availability

The authors will consider any reasonable requests to access the data.
